# Geographical Disparities in Screening and Cancer-Related Health Behaviour

**DOI:** 10.3390/ijerph17041246

**Published:** 2020-02-14

**Authors:** Belinda C. Goodwin, Arlen K. Rowe, Fiona Crawford-Williams, Peter Baade, Suzanne K. Chambers, Nicholas Ralph, Joanne F. Aitken

**Affiliations:** 1 Cancer Council Queensland, 553 Gregory Terrace, Fortitude Valley QLD 4006, Australia; peterbaade@cancerqld.org.au (P.B.); nicholasralph@cancerqld.org.au (N.R.); joanneaitken@cancerqld.org.au (J.F.A.); 2 Institute for Resilient Regions, University of Southern Queensland, Springfield QLD 4300, Australia; arlen.rowe@usq.edu.au (A.K.R.); fiona.crawford-williams@usq.edu.au (F.C.-W.); Suzanne.Chambers@uts.edu.au (S.K.C.); 3 School of Psychology, University of Southern Queensland, Springfield QLD 4300, Australia; 4 Menzies Institute of Health Queensland, Griffith University, Gold Coast QLD 4215, Australia; 5 School of Mathematical Sciences, Queensland University of Technology, Brisbane QLD 4000, Australia; 6 Faculty of Health, University of Technology Sydney, Ultimo NSW 2007, Australia; 7 Exercise Medicine Research Institute, Edith Cowan University, Joondalup WA 6027, Australia; 8 School of Nursing & Midwifery, University of Southern Queensland, Toowoomba QLD 4370, Australia; 9 School of Public Health, The University of Queensland, St Lucia, QLD 4702, Australia

**Keywords:** geographical disparity, health behavior, cancer, public health, diet exercise, alcohol, smoking, cancer screening, regional, remote

## Abstract

This study aimed to identify whether cancer-related health behaviours including participation in cancer screening vary by geographic location in Australia. Data were obtained from the 2014–2015 Australian National Health Survey, a computer-assisted telephone interview that measured a range of health-related issues in a sample of randomly selected households. Chi-square tests and adjusted odds ratios from logistic regression models were computed to assess the association between residential location and cancer-related health behaviours including cancer screening participation, alcohol consumption, smoking, exercise, and fruit and vegetable intake, controlling for age, socio-economic status (SES), education, and place of birth. The findings show insufficient exercise, risky alcohol intake, meeting vegetable intake guidelines, and participation in cervical screening are more likely for those living in inner regional areas and in outer regional/remote areas compared with those living in major cities. Daily smoking and participation in prostate cancer screening were significantly higher for those living in outer regional/remote areas. While participation in cancer screening in Australia does not appear to be negatively impacted by regional or remote living, lifestyle behaviours associated with cancer incidence and mortality are poorer in regional and remote areas. Population-based interventions targeting health behaviour change may be an appropriate target for reducing geographical disparities in cancer outcomes.

## 1. Introduction

A well-documented health divide exists between major cities and regional and remote areas in Australia [1,2,3,4]. Living outside of a major city is associated with higher mortality from a number of chronic health conditions such as diabetes, cardiovascular disease, and cancer [4,5,6,7,8]. For example, the Australian Cancer Atlas (https://atlas.cancer.org.au/) reports a disproportionate burden of some cancers outside of major cities, including higher incidence of lung, cervical, and head and neck cancer and poorer survival from bowel, lung, prostate, skin, and head and neck cancer [5,9]. Poorer quality of life and psychosocial wellbeing is also evident for cancer survivors living outside of major cities [10].

It is well established that alcohol consumption [11], poor diet, limited exercise [12,13,14], and tobacco smoking [15,16] are related to the increased risk of many cancers, as well as poorer outcomes after diagnosis. In Australia, tobacco use contributes 22% of the nation’s cancer burden, while alcohol use contributes a further 3.3% [17]. Whiteman and colleagues, 2015 estimate that up to 37,000 preventable cancers are caused each year by tobacco smoking, alcohol, obesity, and poor diet [18]. Routine population screening for breast, cervical, and bowel cancer in Australia results in earlier detection and increased survival [19,20,21,22], and there is evidence that regular skin examinations and risk-appropriate screening through prostate-specific antigen (PSA) testing in men may reduce mortality from melanoma and prostate cancer, respectively [23,24].

Several potential explanations for geographic disparities in cancer outcomes include populations with larger proportions of older people, social and economic disadvantage, and poorer access to health care outside major cities [25,26,27]. It has been speculated, however, that geographical disparities in cancer outcomes may be partly explained by differences in health behaviours across varying environmental contexts [3]. International patterns of geographic disparities in cancer-related behaviours are mixed. For example, several studies have shown that tobacco smoking [17,28,29] and alcohol consumption [30] are higher in rural and remote areas, whereas others have found no effect or a negative relationship with remoteness [31,32,33]. Living in regional and remote areas has been associated with unhealthier diet and decreased exercise in some studies [31,33,34]. This is consistent with Australian figures that show rates of daily smoking, overweight or obesity, lower levels of exercise and riskier alcohol consumption are higher in regional and remote areas than in major cities [35]. However, other reports suggest that regional and remote residents demonstrate better or equally healthy eating habits than those in major cities [36,37]. It has also been suggested that participation in breast, cervical, and bowel cancer screening is lower in regional and remote areas than in major cities [38,39].

Investigations into geographic disparities in health behaviours have been limited by some key elements. For example, much of the literature on disparities in health behaviours is focused on younger adults (i.e., <50) [33,36,37]. Furthermore, many of the studies do not separate regional areas from rural or remote areas, collapsing all non-metropolitan residents into a “rural” group. In countries with large land masses (e.g., Australia, Canada, and the United States), non-metropolitan residents often fall into two or more distinct categories. For example, a large proportion of residents live outside major city boundaries in “inner regional” areas that are moderately accessible, but often have higher proportions of older, socio-economically disadvantaged residents [40]. Evidence for geographic differences in health behaviours in Australia is inconsistent and most studies are limited in that they fail to control for potential socio-demographic confounders including age, education, and socio-economic status (SES) [40]. This study aimed to identify whether health behaviours known to be associated with cancer risk vary across geographic locations, and to what extent these differences can be explained by socio-demographic factors. Specifically, the study assessed whether, when controlling for potential demographic confounders such as age, education, SES, and place of birth, there are statistically significant geographical disparities in the following:

(1) Rates of screening for breast, cervical, bowel, skin, and prostate cancer.
(2) The likelihood of participating in healthy lifestyle behaviours (i.e., avoiding smoking and alcohol consumption and meeting national guidelines for exercise and fruit and vegetable intake).

The findings can help to appropriately identify intervention targets for addressing the geographical health divide in Australia, particularly in terms of cancer-related outcomes.

## 2. Methods

### 2.1. Data Source

Data were obtained from the 2014–2015 Australian National Health Survey [41], a computer-assisted telephone interview designed to measure a range of health-related issues in a representative sample of randomly selected Australian households. The stratified sampling design ensured that individuals from varying levels of remoteness across all states and territories within Australia took part in the survey. One adult (i.e, 18 years or older) was surveyed from each selected household (*n* = 14,560) Further details on recruitment and sampling procedures are publicly available on the Australian Bureau of Statistics (ABS) website [41]. Access and use of this data for the specific purposes of this study were granted by the ABS based on approval from the university’s Human Research Ethics Committee (ref. H17REA152).

### 2.2. Measures

#### 2.2.1. Demographics

The age in years and biological sex of each participant were recorded. Highest level of education was measured on a five-point scale ranging from “year 8 or below” to “year 12 or higher”. Participants were also coded according to whether or not they were born in Australia.

#### 2.2.2. Remoteness

Level of remoteness of living was based on the Australian Standard Geographical Classification—Remoteness Area (ASGC-RA; Australian Government Department of Health and Ageing, 2011a) [42] The ASGC—RA system codes individual’s residence as either major city (*N* = 9628), inner regional (*N* = 2678), and outer/remote (including outer regional, remote, and very remote, *N* = 2254). Categorisations are based on an index derived from road distance to nearest service centre and population size (for technical details, refer to the ABS, 2011 [42]).

#### 2.2.3. Socio-Economic Status

The Socio-Economic Index for Areas (SEIFA) was applied, whereby each participant is allocated an SES ranking based on their street address (i.e., statistical area level 1; described in ABS, 2011b [43]). A rank of 1 on the SEIFA indicates that the participant resides in an area assigned the lowest SES and a rank of 10 reflects the highest SES areas. The SES variable was treated as a continuous numeric variable in the main analysis.

#### 2.2.4. Risky Alcohol Consumption

Participants were asked a series of detailed questions regarding the frequency and amount of alcohol consumption in the previous 12 months (for details, refer to ABS, 2017 [41]). On the basis of their responses, participants were coded according to whether they exceeded the 2009 National Health and Medical Research Council (NHMRC) Guidelines to Reduce Health Risks from Drinking Alcohol [44] of consuming, on average, more than two standard drinks per day. A binary yes/no “risky alcohol use” variable was created from this.

#### 2.2.5. Daily Smoker

Participants were asked to select their smoking status from five options; current daily smoker, current weekly smoker, current smoker (other), ex-smoker, and never-smoked. A binary yes/no variable was created from this to reflect whether the respondent was a current daily smoker or not.

#### 2.2.6. Fruit and Vegetable Intake

Participants were asked to report the number of serves of fruit and serves of vegetables they typically ate each day. Details regarding the definition of a “serve” were presented to each participant (e.g., 1/2 cup cooked green or orange vegetables and 1 medium apple, banana, orange or pear) [41]. On the basis of the 2013 NHMRC Australian Dietary Guidelines [45], two binary variables were created that reflect whether participants typically consumed five or more serves of vegetable per day (yes/no), and two or more serves of fruit (yes/no).

#### 2.2.7. Exercise

On the basis of a series of detailed questions regarding the participant’s leisure time exercise in the previous week, it was determined whether participants met Australia’s Physical Activity and Sedentary Behaviour Guidelines for Adults [46] (for technical details, refer to ABS, 2017 [41]. These guidelines state that adults should accumulate 150 to 300 min of moderate intensity physical activity or 75 to 150 min of vigorous intensity physical activity (or a combination thereof each week). It is also recommended that muscle strengthening training is undertaken on at least two days per week. Detailed definitions of “intensity” were presented to each participant (e.g., moderate intensity exercise was defined as activities that caused a moderate increase in the heart rate or breathing of the respondent) [41]. A binary yes/no “meets physical activity (PA) guidelines” variable was created that reflected whether participants’ physical activity habits fulfilled these recommendations.

#### 2.2.8. Cancer Screening

Participants were asked whether they had undergone a screening test for breast cancer (e.g., mammogram), cervical cancer (e.g., pap smear), and bowel cancer (e.g., fecal occult blood test) within the previous two years—the recommended frequency for the early detection of breast, bowel, and cervical cancer at the time of data collection for the relevant age groups (described below) [47]. Participants were also asked whether they had undergone screening for prostate cancer in the last two years (males only) and whether they regularly checked their skin for any changes in freckles or moles. Although there are currently no population-based screening programs for prostate or skin cancer screening in Australia, efforts to detect these cancers early are encouraged through regular skin examinations and risk-appropriate PSA testing, respectively [24,48,49]. Where screening guidelines apply, only participants within recommended the age groups for each type of screening were included in the analysis using the cancer screening variables (i.e., breast: 50–74-year-old females, cervical: 18–69-year-old females, bowel: 50–74-year old males and females). Males over 50 and all adults were included in the analyses regarding prostate and skin cancer screening, respectively.

## 3. Analysis

A series of chi-square analyses were conducted to assess whether the likelihood of engaging in each form of cancer screening, daily smoking, risky alcohol intake, and meeting recommended health guidelines varied across geographic locations. Pearson’s bi-serial correlations were conducted to identify significant bivariate relationships between binary and continuous demographic variables including gender, age, education, SES, and country of birth and screening and health behaviour outcome variables. Multivariate binary logistic regression models were then conducted to assess geographic disparities controlling for significant demographic predictors of behaviour (i.e., age, education, SES, and place of birth). Where significant geographical disparities were evident (based on significant chi-square statistics), screening and health behaviour outcomes were compared between regional and major city and outer/remote and major city groups. Analyses were conducted using SPSS Version 24. A Bonferroni adjustment [50] was applied to reduce the probability of type I errors, whereby 0.05 was divided by the number of tests carried out in the main analysis (*n* = 20), Only *p*-values below the critical value of 0.0025 were interpreted as statistically significant.

## 4. Results

### 4.1. Sample Characteristics

The final sample consisted of 14,560 adults ranging between 18 and 85 years of age (M = 49.12, SD = 17.61). Table 1 shows the percentage of participants in each sex, age, education, country of birth, remoteness, and SES category. The distributions of participants that were male (46.1%) and female (53.9%), from varying SES indices, born in Australia (69.5%), educated at a year 12 level or lower (66.1%), and living in a major city (66.1%) were similar to that of the broader Australian population [51].

A bivariate analysis of variance showed that the mean age of participants differed significantly across major city (M = 48.18, SD = 17.53), inner regional (M = 51.98, SD = 17.93), and outer regional/remote (M = 49.73, SD = 17.16) areas, F (14,559.2) = 50.65, *p <* 0.001. As shown in Table 1, the demographic characteristics of each geographical group differed significantly in terms of SES, highest education level, and country of birth. For example, higher proportions of inner regional (31.3%) and outer/remote (26.6%) participants were from the lowest SES bracket when compared with major cities (13.4%). Furthermore, a higher proportion of participants in major cities reported having completed year 12 (61.2%) when compared with those in inner regional (38.5%) or outer/remote (40.7%) areas and a lower proportion of those in major cities reported being born in Australia (62.9%) when compared with those in inner regional (84.2%) or outer/remote areas (80.2%).

### 4.2. Screening

Several weak associations between demographic variables and cancer screening were identified. Females were slightly more likely to report screening for bowel cancer (*r* = 0.062, *p* < 0.001) and regular skin checks (*r* = 0.070, *p* < 0.001). Older age was associated with prostate cancer screening (*r* = 0.137, *p* < 0.001) and skin checks (*r* = 0.183, *p* < 0.001), and those higher in SES areas were more likely to report all forms of cancer screening (*r* = −0.22, *p* = 0.008 to *r* = −0.09, *p* < 0.001). A higher level of education was weakly associated with breast (*r* = 0.084, *p* < 0.001), cervical, (*r* = 0.110, *p* < 0.001), and bowel (*r* = 0.057, *p* < 0.000) cancer screening and skin checks (*r* = 0.055, *p* < 0.001), and participants who were born in Australia were slightly more likely to report prostate (*r* = 0.071, *p* < 0.001) and bowel (*r* = 0.042, *p* < 0.001) cancer screening and skin checks (*r* = 0.200, *p* < 0.001).

There was a significant bivariate association between geographical remoteness and both prostate cancer screening (χ^2^ (2) = 9.30, *p* = 0.010) and skin checks (χ^2^ (2) = 107.88, *p* < 0.001) (Table 2). After adjustment for age, education, SES, and country birth, these significant associations remained, along with an association with cervical screening (χ^2^ (2) = 11.89, *p* = 0.003). Respondents living in inner regional areas were more likely to have undergone prostate cancer (odds ratio (OR)_adj_ = 1.20, 1.05–1.38) and cervical screening (OR_adj_ = 1.39, 1.12–1.70) in the last two years than those living in major cities.

### 4.3. Health Behaviours

Significant associations between demographic variables and meeting health recommendations were identified in almost all cases. Males were slightly more likely to report risky alcohol intake (*r* = 0.139, *p* = < 0.001), daily tobacco smoking (*r* = −0.056, *p* = < 0.001), and meeting exercise guidelines (*r* = −0.023, *p* = < 0.001), and females were slightly more likely to report meeting recommended fruit (*r* = 0.106, *p* = < 0.001) and vegetable (*r* = 0.040, *p* = < 0.001) intake guidelines. Younger participants were more likely to report risky alcohol intake (*r* = −0.352, *p* = < 0.001), daily tobacco smoking (*r* = −0.104, *p* = < 0.001), and meeting exercise guidelines (*r* = −0.026, *p* = < 0.001), and older participants were more likely to report meeting recommended fruit (*r* = 0.122, *p* = < 0.001) and vegetable (*r* = 0.035, *p* = < 0.001) intake guidelines. Higher SES was associated with risky alcohol intake (*r* = 0.056, *p* = < 0.001), and meeting exercise (*r* = 0.084, *p* = < 0.001) and fruit intake (*r* = 0.050, *p* = < 0.001) guidelines, while lower SES was associated with daily smoking (*r* = −0.142, *p* = < 0.001). Higher levels of education were associated with daily smoking (*r* = 0.106, *p* = < 0.001), and lower levels of education associated with risky alcohol intake (*r* = −0.136, *p* = < 0.001) and meeting exercise guidelines (*r* = −0.064, *p* = < 0.001). Participants born outside Australia were slightly more likely to report risky alcohol intake (*r* = 0.059, *p* = < 0.001) and meeting fruit intake guidelines (*r* = 0.046, *p* = < 0.001), while those born in Australia were slightly more likely to report being a daily smoker (*r* = −0.082, *p* = < 0.001) and meeting vegetable intake guidelines (*r* = −0.029, *p* = < 0.001).

On the basis of bivariate analyses, geographic disparities were evident for all health behaviours including risky alcohol intake (χ^2^ (2) = 11.37, *p* < 0.001), daily smoking (χ^2^ (2) = 95.33, *p* < 0.001), meeting exercise guidelines (χ^2^ (2) = 17.66, *p* < 0.001), and fruit (χ^2^ (2) = 9.13, *p* = 0.010,) and vegetable (χ^2^ (2) = 42.49, *p* < 0.001) intake guidelines. As shown in Table 3, after adjustment for age, education, SES, and country of birth, significant associations with daily smoking, alcohol, and vegetable intake remained, however, associations with meeting exercise guidelines (χ^2^ (2) = 2.69, *p* = 0.261) and fruit intake guidelines (χ^2^ (2) = 3.51, *p* = 0.173) were no longer significant. According to contrasts, those living in inner regional areas were more likely than those in major cities to report risky alcohol intake (OR_adj_ = 1.22, 1.10–1.35) and meeting vegetable intake guidelines (OR_adj_ = 1.21, 1.04–1.41). In addition, participants in outer/remote areas were more likely than those in major cities to report risky alcohol intake (OR_adj_ = 1.19, 1.07–1.33), daily smoking (OR_adj_ = 1.27, 1.12–1.44), and meeting vegetable intake guidelines (OR_adj_ = 1.60, 1.38–1.85).

## 5. Discussion

Understanding the health behaviours of those living in different geographic regions may provide some insight into a well-documented geographical divide in health outcomes. In particular, these findings support suggestions that geographical disparities in cancer outcomes are in part because of geographical disparities in cancer-related health behaviors [3], providing a plausible partial explanation for higher cancer incidence and mortality rates outside of major cities in Australia [5,9].

According to current findings, cancer screening is just as common, if not more so, in regional Australia compared with metropolitan areas and geographical differences in cancer screening rates are largely unaffected by geographic variance in demographic factors. These findings conflict somewhat with previous studies [38,39,52], although some previous research does suggest that rural women are no less likely than those living in metropolitan areas to attend mammography [53,54]. Potentially, the relatively equal screening rates across geographic areas may reflect the success of community-based screening campaigns concentrated in rural populations such as the mobile BreastScreen Australia bus [55] and Rotary BowelScan [56]. That is, the intermittent availability of health services in regional and remote areas [57] may result in heavier promotion of screening services when they do visit regional communities, providing stronger urgency and impetus to participate when available.

In terms of health behaviours that can help to reduce cancer risk, geographic disparities were evident with both inner regional and outer regional/remote residents reporting higher rates of alcohol intake and daily smoking. These differences were not explained by the varying demographic make-up of each geographical area in terms of age, SES, education, and country of birth. Generally, demographic associations in the current findings provide some support for the notion that poorer health behaviours are more common among the socially and economically disadvantaged because of several factors including higher work/stress loads, reduced time and monetary resources, and a poorer understanding of health [58,59,60], but in the case of alcohol and tobacco smoking, these factors do not account for regional disparities. Efforts to decrease risky alcohol intake and daily smoking clearly need to target issues specific to social and economic disadvantage [61]. However, as suggested by previous research, in addressing regional disparities in alcohol and tobacco use, public health initiatives may need to consider other cultural or environmental factors unique to regional and remote communities [62].

The tendency for those living further away from major cities to be less likely to meet exercise and fruit intake guidelines did appear to be explained by variation in the demography of each area. For example, not meeting exercise and fruit intake guidelines was associated with lower levels of SES and education; both more common in inner regional and outer/remote areas. The reasons for poor dietary intake and low physical activity may be attributed to the following: ongoing challenges in implementing community-wide physical activity and dietary intake promotional campaigns in socio-economical disadvantaged regional areas; social isolation; reduced opportunity to access resources to increase physical activity; environmental barriers including extreme weather; poor infrastructure including a lack of footpaths and lighting; and the need to drive to shops and services [63,64,65]. However, gaps remain in the chain of evidence between population-based efforts to improve physical activity and dietary intake and the effect of such interventions in reducing the disparity in outcomes among regional and rural individuals with cancer [66,67].

## 6. Strengths and Limitations

The main strength of the study is the large, representative sample utilising stratified random sampling, including adequate subsample sizes, allowing for the examination of differences in health behaviours across geographic regions, including metropolitan and varying levels of remoteness. The examination of such differences, while controlling for well-known socio-demographic factors, uniquely contributes to the existing literature. The data used in this study were self-reported and not able to be independently verified, thus we are unable to exclude potential misclassification and possible bias due to differential misreporting of lifestyle factors such as alcohol consumption. The national Health Survey Response rate is over 80% [41]; thus, although a potential effect of non-respondent bias cannot be ruled out, it is likely to be small.

Although the results of the current study provide insight into associations between residential location and cancer-related health behaviours, future investigation is needed to determine the potential for behaviour change interventions to reduce geographic disparities in cancer incidence. It is also acknowledged that associations between demographic variables and cancer-related health behaviours were for the most part weak and, given the large sample size, should be interpreted with caution.

## 7. Conclusions

Living in a regional or remote location does not appear to be a barrier to cancer screening participation in Australia. There are geographic disparities in other health behaviours known to be associated with cancer incidence and mortality including alcohol consumption, smoking, fruit and vegetable intake, and exercise. Improving these behaviours on a population level may be an appropriate target for reducing geographical disparities in cancer outcomes. In particular, public health interventions aimed at changing environmental factors to ensure that healthy behaviour is promoted and facilitated in regional and remote areas should be a key focus. The findings provide a basis and direction for future research to investigate casual links between geographical disparities in health behaviour and geographical disparities in cancer outcomes.

## Figures and Tables

**Table 1:** Demographic characteristics according to remoteness level.

	Major City n (%)	Inner Regional n (%)	Outer & Remote n (%)	Total n (%)	*X^2^*
**Sex**					4.58
Female	5199 (53.9%)	1504 (56.2%)	1211 (53.7%)	7907 (54.7)	
Male	4436 (46.1%)	1174 (43.8%)	1043 (46.3%)	6653 (45.3%)	
**Age bracket**					113.23 *
18–25	917 (69.8%)	221 (16.8%)	175 (13.3%)	1313 (9.0%)	
26–49	4360 (69.6%)	959 (15.2%)	961 (15.2%)	6310 (38.4%	
50–74	3483 (62.3%)	1191 (21.3%)	914 (16.4%)	5588 (38.4%)	
75+	838 (62.1%)	307 (22.8%)	204 (15.1%)	1349 (9.3%)	
**SES Quintile**					1341.02 *
1st (lowest)	1294 (13.4%)	837 (31.3%)	600 (26.6%)	2731 (18.8%)	
2nd	1700 (17.7%	657 (24.5%)	546 (24.2%)	2903 (19.9%)	
3rd	1767 (18.4%)	660 (24.6%)	570 (25.3%)	2997 (20.6%)	
4th	2238 (23.2%)	319 (11.9%)	357 (15.8%)	2914 (20.0%)	
5th (highest)	2629 (27.3%)	205 (7.7%)	181 (8.0%)	3015 (20.7%)	
**Education**					620.18 *
Year 12 or higher	5890 (61.2%)	1030 (38.5%)	918 (40.7%)	7838 (53.5%)	
Year 9–11	3128 (32.5%)	1390 (51.9%)	1131 (50.2%)	5649 (38.8%)	
Year 8 or below	610 (6.3%)	258 (9.6%)	205 (9.1%)	1073 (7.4%)	
**Country of birth**					500.86 *
Australia	6056 (62.9%)	2255 (84.2%)	1808 (80.2%)	10,119 (69.5%)	
United Kingdom	818 (8.5%)	178 (6.6%)	132 (5.9%)	1128 (7.7%)	
South-eastern Europe	176 (1.8%)	10 (0.4%)	23 (1.0%)	209 (1.4%)	
New Zealand	273 (2.8%)	41 (1.5%)	49 (2.2%)	363 (2.5%)	
India	246 (2.6%)	16 (0.6%)	22 (7.7%)	284 (2.0%)	
Sub-Saharan Africa	188 (2.0%)	17 (0.6%	25 (0.2%	230 (1.6%)	
North-west Europe	163 (1.7%)	26 (1.0%)	40 (1.8%)	229 (1.6%)	
Other	1708 (17.7%)	135 (5.0%)	155 (7.8%)	1056 (11.4%)	
**Total**	9628 (66.1%)	2678 (18.4%)	2254 (15.5%)	14,560 (100%)	

Note: Socio-Economic Index for Areas (SEIFA) quintiles and collapsed education variable reported in table for brevity. SES, socioeconomic status. * = *p* < 0.001.

**Table 2:** Association between geographic remoteness and self-reported participation in screening activities.

	Breast Screening (Females, 50–74 Years *n* = 2974)			Cervical Screening (Females, 18–69 Years *n* = 6585)			Prostate Cancer Screening (Males, 50–74 Years *n* = 2620)			Bowel Cancer Screening (Adults, 50–74 Years *n* = 5588)			Skin Cancer Screening (Adults, 18+ Years *n* = 14,650)		
	N (%) ^1^	OR ^2^	OR_ADJ_^3^	N (%) ^1^	OR ^2^	OR_ADJ_^3^	N (%) ^1^	OR ^2^	OR_ADJ_^3^	N (%) ^1^	OR ^2^	OR_ADJ_^3^	N (%) ^1^	OR ^2^	OR_ADJ_^3^
Major city ^4^	1040 (55.2%)	1.00	1.00	2110 (%)	1.00	1.00	619 (38.6%)	1.00	1.00	1104 (31.7%)	1.00	1.00	5605 (58.2%)	1.00	1.00
Inner regional	340 (53.0%)	0.91 (0.76–1.09)	1.04 (86–1.26)	569 48.3%)	1.01 (0.88–1.14)	1.20 (1.05–1.38)	251 (45.6%)	1.45 (1.45–1.46)	1.39 (1.12–1.71)	401 (33.7%)	1.06 (0.99–1.15)	1.05 (0.88–1.27)	1823 (68.1%)	1.53 (1.40–1.68)	1.31 (1.18–1.44)
Outer/Remote	246 (54.8%)	0.98 (0.80–1.21)	1.15 (0.93–1.43)	508 (49.2%)	1.04 (0.91–1.19)	1.22 (1.06–1.40)	176 (37.8%)	1.46 (1.16–1.17)	1.04 (0.83–1.31)	309 (33.8%)	1.04 (0.93–1.15)	1.06 (0.87–1.30)	1479 (65.6%)	1.37 (1.25–1.51)	1.26 (1.14–1.40)
LHR test ^5^		χ^2^ = 1.00 df = 2, *p* = 0.607	χ^2^ = 1.69 df = 2, *p* = 0.430		χ^2^ = 0.30 df = 2, *p* = 0.860	χ^2^ = 11.89 df = 2, *p* = 0.003		χ^2^ = 9.30 df = 2, *p* = 0.010	χ^2^ = 9.66 df = 2, *p* = 0.002		χ^2^ = 2.48 df = 2, *p* = 0.290	χ^2^ = 0.43 df = 2, *p* = 0.805		χ^2^ = 107.88 df = 2, *p* < 0.001	χ^2^ = 38.40 df = 2, *p* < 0.001

^1^ N = Number of eligible respondents who responded “yes” to the item, (%) = percentage of eligible respondents in this remoteness category who responded “yes” to the item. ^2^ Unadjusted odds ratios (ORs) of participating in screening activities (self-reported). ^3^ Adjusted odds ratios (OR_ADJ_) from multivariable logistic regression models, adjusted for age, SES, education, and whether born in Australia. ^4^ Reference category for odds ratios. ^5^ Likelihood ratio (LHR) test, based on the chi-squared statistic, for the significance of the remoteness variable.

**Table 3:** Association between geographic remoteness and self-reported health behaviours (*N* = 14,650).

	Risky Alcohol (Adults, 18+ Years)			Daily Smoker (Adults, 18+ Years)			Meets Exercise Guidelines (Adults, 18+ Years)			2+ Fruit per Day (Adults, 18+ Years)			5+ Vegetables per Day (Adults, 18+ Years)		
	N (%) ^1^	OR^2^	OR_ADJ_^3^	N (%) ^1^	OR ^2^	OR_ADJ_^3^	N (%) ^1^	OR ^2^	OR_ADJ_^3^	N (%) ^1^	OR ^2^	OR_ADJ_^3^	N (%) ^1^	OR ^2^	OR_ADJ_^3^
Major city ^4^	3422 (35.5%)	1.00	1.00	1298 (13.5%)	1.00	1.00	1056 (15.8%)	1.00	1.00	4957 (51.5%)	1.00	1.00	848 (8.8%)	1.00	1.00
Inner regional	1012 (37.8%)	1.10 (1.01–1.20)	1.22 (1.10–1.35)	462 (17.3%)	1.34 (1.19–1.50)	0.95 (0.84–1.08)	371 (13.9%)	0.85 (0.76–0.97)	1.02 (0.90–1.16)	1367 (51.0%)	0.98 (0.90–1.70)	1.06 (0.97–1.16)	283 (10.6%)	1.22 (1.06–1.41)	1.213 (1.04–1.41)
Outer/Remote	877 (38.9%)	1.16 (1.05–1.27)	1.19 (1.07–1.33)	481 (21.3%)	1.74 (1.55–1.96)	1.27 (1.12–1.44)	286 (12.7%)	0.77 (0.67–0.88)	0.90 (0.78–1.03)	1081 (48.0%)	0.87 (0.79–0.95)	95 (0.86–1.047)	298 (13.2%)	1.58 (1.37–1.82)	1.598 (1.38–1.85)
LHR test ^5^		χ^2^ = 11.37 df = 2, *p* = (0.003)	χ^2^ = 20.12 df = 2, *p* = (<0.001)		χ^2^ = 95.33 df = 2, *p* = (<0.001)	χ^2^ = 18.60 df = 2, *p* = (<0.001)		χ^2^ = 17.66 df = 2, *p* = (<0.001)	χ^2^ = 2.69 df = 2, *p* = (261)		χ^2^ = 9.13 df = 2, *p* = (0.010)	χ^2^ = 3.51 df = 2, *p* = (173)		χ^2^ = 42.29 df = 2, *p* = (<0.001)	χ^2^ = 39.30 df = 2, *p* = (<0.001)

^1^ N = Number of eligible respondents who met the criteria for this item, (%) = percentage of eligible respondents in this remoteness category who met the criteria for this item ^2^ Unadjusted odds ratios (ORs) of participating in screening activities (self-reported). ^3^ Adjusted odds ratios (OR_ADJ_) from multivariable logistic regression models, adjusted for age, SES, education, and whether born in Australia. ^4^ Reference category for odds ratios. ^5^ Likelihood ratio test, based on the chi-squared statistic, for the significance of the remoteness variable.
